# Transcription factor Zbtb1 interacts with bridging factor Lmo2 and maintains the T-lineage differentiation capacity of lymphoid progenitor cells

**DOI:** 10.1016/j.jbc.2022.102506

**Published:** 2022-09-17

**Authors:** Maria Koizumi, Yuichi Kama, Ken-ichi Hirano, Yusuke Endo, Tomoaki Tanaka, Katsuto Hozumi, Hiroyuki Hosokawa

**Affiliations:** 1 Department of Immunology, Tokai University School of Medicine, Isehara, Kanagawa, Japan; 2 Laboratory of Medical Omics Research, Kazusa DNA Research Institute, Chiba, Japan; 3 Department of Omics Medicine, Graduate School of Medicine, Chiba University, Chiba, Japan; 4 Department of Molecular Diagnosis, Graduate School of Medicine, Chiba University, Chiba, Japan; 5 Institute of Medical Sciences, Tokai University, Isehara, Kanagawa, Japan

**Keywords:** hematopoietic stem cell, hematopoiesis, lymphocyte, T-cell, cell differentiation, gene regulation, transcription factor, Zbtb1, Lmo2, BM, bone marrow, ChIP-seq, chromatin immunoprecipitation followed by deep sequencing, DEG, differentially expressed gene, DN, double-negative, GO, Gene Ontology, HPC, hematopoietic progenitor cell, HSC, hematopoietic stem cell, HSPC, hematopoietic stem and progenitor cell, ILC, innate lymphoid cell, LP, lymphoid progenitor, sgRNA, single-guide RNA, TPM, transcripts per kilobase million

## Abstract

Hematopoietic stem and progenitor cells can differentiate into all types of blood cells. Regulatory mechanisms underlying pluripotency in progenitors, such as the ability of lymphoid progenitor cells to differentiate into T-lineage, remain unclear. We have previously reported that LIM domain only 2 (Lmo2), a bridging factor in large transcriptional complexes, is essential to retain the ability of lymphoid progenitors to differentiate into T-lineage. However, biochemical characterization of Lmo2 protein complexes in physiological hematopoietic progenitors remains obscure. Here, we identified approximately 600 Lmo2-interacting molecules in a lymphoid progenitor cell line by two-step affinity purification with LC-MS/MS analysis. Zinc finger and BTB domain containing 1 (Zbtb1) and CBFA2/RUNX1 partner transcriptional corepressor 3 (Cbfa2t3) were found to be the functionally important binding partners of Lmo2. We determined CRISPR/Cas9-mediated acute disruption of *Zbtb1* or *Cbfa2t3* in the lymphoid progenitor or bone marrow–derived primary hematopoietic progenitor cells causes significant defects in the initiation of T-cell development when Notch signaling is activated. Our transcriptome analysis of *Zbtb1*- or *Cbfa2t3*-deficient lymphoid progenitors revealed that *Tcf7* was a common target for both factors. Additionally, ChIP-seq analysis showed that Lmo2, Zbtb1, and Cbfa2t3 cobind to the *Tcf7* upstream enhancer region, which is occupied by the Notch intracellular domain/RBPJ transcriptional complex after Notch stimulation, in lymphoid progenitors. Moreover, transduction with *Tcf7* restored the defect in the T-lineage potential of *Zbtb1*-deficient lymphoid progenitors. Thus, in lymphoid progenitors, the Lmo2/Zbtb1/Cbfa2t3 complex directly binds to the *Tcf7* locus and maintains responsiveness to the Notch-mediated inductive signaling to facilitate T-lineage differentiation.

T-cell development is initiated by Notch signaling when prethymic lymphoid progenitors (LPs) migrate into the thymus (1, 2, 3). LPs express Notch receptors, Notch1 and Notch2, on their surface, and the thymic epithelial cells provide a Notch-ligand, Delta-like 4 (DLL4), to trigger the T-lineage developmental program (4, 5, 6, 7, 8). The dominant roles of Notch signal in the initiation of T-lineage program have been well studied using *in vitro* T-cell culture systems with conditional or CRISPR/Cas9-mediated disruption of Notch receptors (4, 9, 10). The similarities in the gene expression profiles of *in vitro* generated T progenitors and their *in vivo* counterparts have been validated *via* transcriptomic analysis (11, 12). Notch-dependent progression of T progenitor stages is regulated by the timely activation and repression of transcription factors (10, 13, 14). Transcription factor 7 (*Tcf7*, encoding the TCF1 protein) and GATA-binding protein 3 (*Gata3*) are the earliest Notch target genes that act as crucial regulatory transcription factors for T-cell specification in the thymus (15, 16, 17, 18).

Hematopoietic stem cells (HSCs) are maintained in the bone marrow (BM) and can differentiate into all types of blood cells. HSCs gradually differentiate into hematopoietic progenitor cells (HPCs) with a limited differentiation potential. LPs (or lymphoid-primed multipotent progenitors) retain the capacity to give rise to not only lymphoid lineage but also myeloid lineage cells (19). A small portion of LPs in the BM migrate into the thymus and become T cells. While the biochemical characteristics of transcription factors in differentiated hematopoietic cells have been extensively examined (20, 21, 22, 23, 24, 25), those in physiological LPs, especially those responsible for maintaining the T-lineage potential, remain elusive. One of the greatest difficulties in handling LPs is their rarity *in vivo*. In addition, recent advances in single-cell multiomics approaches combined with lineage tracing have clearly indicated that HPCs are highly heterogeneous (26, 27). To overcome these problems, LP cell lines derived from EBF transcription factor 1 (*Ebf1*)–deficient HSCs have been established by several different groups (28, 29, 30, 31). *Ebf1*-deficient LP cells are not transformed cells and can be maintained under B-cell conditions with monolayers of stromal cells. They have homogeneous properties of LPs and their ability to differentiate into T-lineage *via* Notch stimulation *in vivo* and *in vitro* (30, 31).

LIM domain only 2 (Lmo2) organizes large transcriptional complexes with basic helix-loop-helix (bHLH) and GATA family members and others in hematological tumors (32, 33). Lmo2 is highly expressed in HSCs and HPCs, and is sharply downregulated in the early stages of T-cell development in the thymus (34). Dysregulation of Lmo2 expression induces T-cell acute lymphoblastic leukemia (35, 36). Reprogramming of hematopoietic stem and progenitor cells (HSPCs) from differentiated blood cells or fibroblasts is induced by the transient expression of combinations of several transcription factors, including Lmo2 (37, 38). These results suggest that Lmo2 is essential for the HSPC reprogramming and is involved in maintaining the pluripotency of HSPCs (39). However, the biochemical characteristics of Lmo2 in physiological HPCs remain unclear.

In our attempt to establish *Ebf1*-deficient LP cell lines, we unexpectedly established LP cell lines with or without T-lineage potential and found Lmo2 to be a transcription factor that is essential for maintaining the potential of LPs to differentiate into T-lineage (31). LP cell lines without T-lineage potential have significantly low *Lmo2* expression, and the ability to differentiate into T cell is abrogated by the disruption of *Lmo2* in LP cell lines with T-lineage potential. In LPs, Lmo2 directly binds to the *Tcf7* locus, one of the earliest Notch target genes, and maintains a poised chromatin configuration for the appropriate activation of *Tcf7*, when Notch signaling is provided (31).

In this study, we performed the proteomics analysis of Lmo2 protein complexes in *Ebf1*-deficient LP cell lines. In addition to previously reported Lmo2-interacting molecules, such as Cbfa2t3, in blood tumors, Zbtb1 is a potential DNA-binding subunit of the Lmo2 complex in LPs. CRISPR/Cas9-mediated deletion of *Zbtb1* or *Cbfa2t3* in *Ebf1*-deficient LPs induced an acute loss of the ability to differentiate into T-lineage cells. Zbtb1 and Cbfa2t3 bound to the upstream region of the *Tcf7* locus, which is co-occupied by Lmo2, and *Zbtb1*- or *Cbfa2t3*-deficient LPs showed decreased *Tcf7* expression. Transduction of *Tcf7* restored the T-lineage differentiation potential of *Zbtb1*-deficient LPs. Finally, we confirmed that the acute disruption of *Zbtb1* or *Cbfa2t3* in BM-derived primary HPCs resulted in a significantly decreased T-lineage potential. Taken together, we identified a novel Lmo2 binding partner, Zbtb1, and demonstrated functional importance of Zbtb1 and Cbfa2t3 in retaining the ability of LPs to differentiate into T-lineages.

## Results

### Zbtb1 is a novel interacting molecule of Lmo2 in LPs

**Figure 1:**
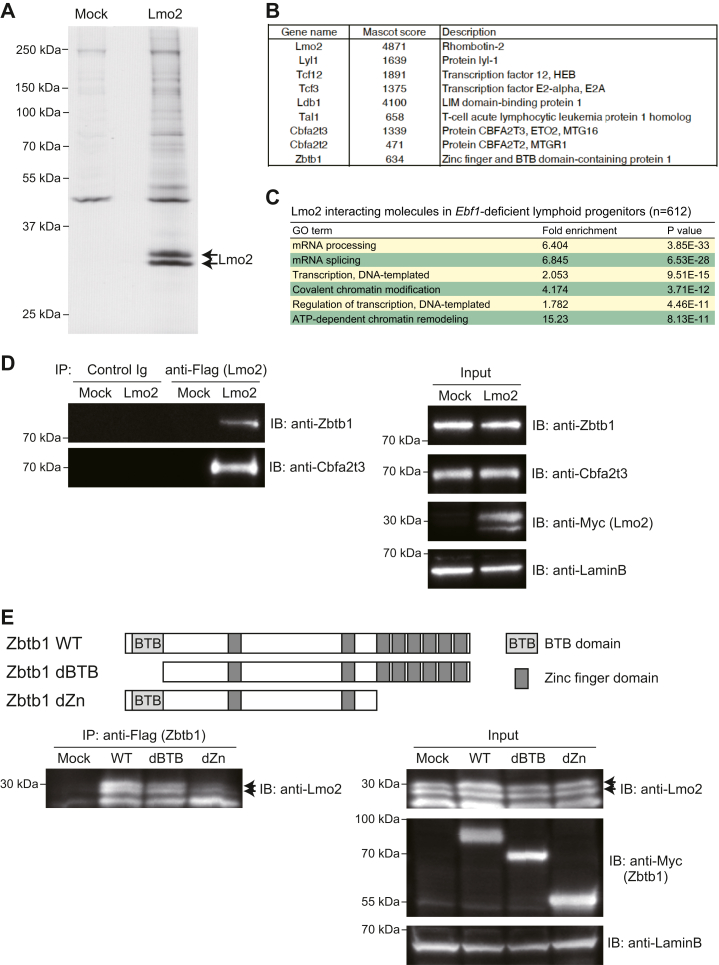
**Identification of Lmo2-interacting molecules in LPs.***A*, Myc- and Flag-tagged Lmo2 was retrovirally transduced into *Ebf1*-deficient LPs. Total extracts from Myc-Flag-Lmo2-expressing LPs were subjected to two-step affinity purification followed by SDS-PAGE and silver staining. All of the visible bands were subjected to mass spectrometry analysis. *B*, representative Lmo2-binding molecules in LPs are shown with Mascot scores. The full list of the Lmo2-binding molecules is shown in Table S1. *C*, Gene Ontology (GO) annotation was performed using the DAVID analysis tool (http://david.ncifcrf.gov/). Top six GO terms for Lmo2-interacting molecules in *Ebf1*-deficient LPs are shown. *D*, total extracts from Mock or Myc-Flag-Lmo2 transduced LPs were subjected to immunoprecipitation (IP) with mouse control Ig or anti-Flag mAbs followed by immunoblotting with anti-Zbtb1, or anti-Cbfa2t3 Abs. Nuclear lysates (input) were also subjected to immunoblotting with anti-Zbtb1, anti-Cbfa2t3, anti-Myc (Lmo2), and anti-LaminB Abs. *E*, Lmo2 and Myc-Flag-tagged Zbtb1 mutants (WT, dBTB, or dZn) were transiently transduced into 293T cells. Total extracts from Lmo2 and Zbtb1 transduced 293T cells were subjected to immunoprecipitation with anti-Flag mAb followed by immunoblotting with anti-Lmo2 mAb. Nuclear lysates (input) were also subjected to immunoblotting with anti-Lmo2, anti-Myc (Zbtb1), and anti-LaminB Abs. Data are representative of two (*D* and *E*) or three (*A*) independent experiments. LP, lymphoid progenitor.

Lmo2 acts as a bridging factor in large transcriptional complexes with transcription factors and chromatin remodeling-related factors (32, 33). To dissect the molecular mechanisms involved in the Lmo2-mediated maintenance of T-lineage potential in LPs, we performed proteomic analysis of Lmo2-interacting molecules. We took advantage of a highly tractable *Ebf1*-deficient LP cell line that retains its potential to differentiate into T-lineage by Notch stimulation *in vivo* and *in vitro* (31). *Ebf1*-deficient LPs were transduced with Myc- and Flag-tagged Lmo2 and subjected to two-step affinity purification followed by SDS-PAGE and silver staining (Fig. 1*A*). Analysis by LC-MS/MS identified more than 600 Lmo2-interacting molecules in physiological LPs, including the previously reported components of Lmo2 complexes in hematological cancer cell lines, Lyl1, Tal1, Ldb1, Tcf12, Tcf3, and Cbfa2t3 (also known as ETO2 or MTG16) (Fig. 1*B* and Table S1) (32, 33). Another member of the ETO family, Cbfa2t2 (MTGR1), was also identified with a relatively lower enrichment score (Fig. 1*B* and Table S1). Gene ontology analysis of Lmo2-interacting molecules showed that the proteins involved in the regulation of transcription and chromatin remodeling were highly enriched (Fig. 1*C*). In addition to the previously reported Lmo2-interacting transcriptional corepressor, Cbfa2t3 (40, 41), the novel Lmo2-associating transcription factor, Zbtb1, is known to play important roles in hematopoiesis and T-cell development (42, 43, 44, 45). Their association with Lmo2 was repeatedly detected and had one of the highest signals in our mass spectrometry analyses (Fig. 1*B* and Table S1). The interactions between Lmo2 and Zbtb1 or Cbfa2t3 were validated *via* coimmunoprecipitation with immunoblotting in *Ebf1*-deficient LPs (Figs. 1*D* and S1*A*), and all three factors are coexpressed in primary common lymphoid progenitors (Fig. S1*B*) (34). Zbtb1 possesses the BTB domain and eight zinc finger domains. Among them, the six C-terminal zinc fingers of Zbtb1 could be involved in the interaction with Lmo2 (Fig. 1*E*). Thus, Zbtb1 and Cbfa2t3 may play important roles in Lmo2-mediated maintenance of T-lineage potential in LP cells.

### Zbtb1 is essential to retain the ability of LPs to differentiate into T-lineage

**Figure 2:**
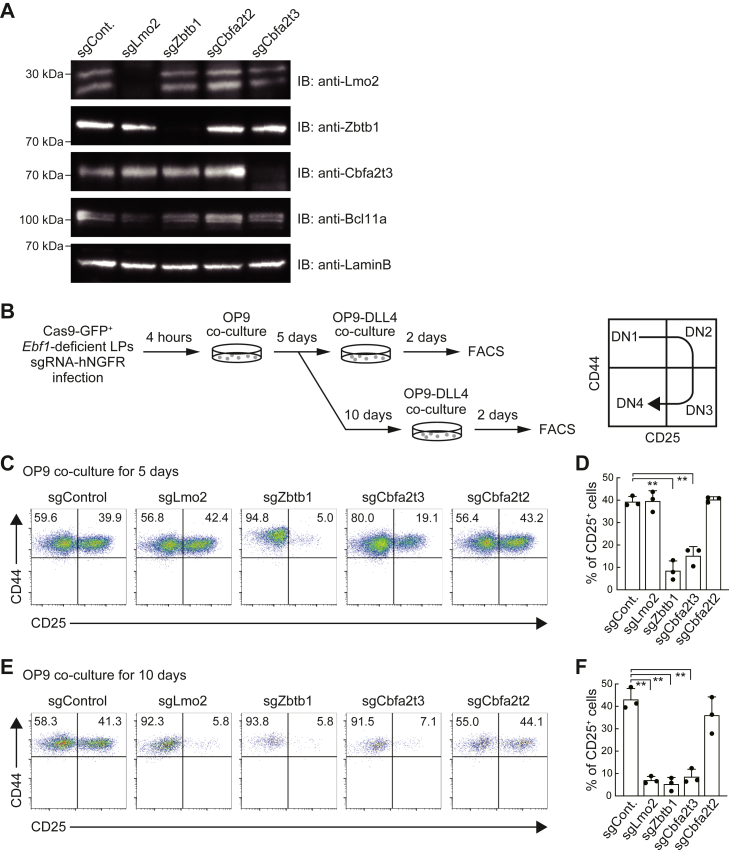
**Loss of *Zbtb1* and *Cbfa2t3* leads to the T-lineage differentiation arrest in LPs.***A*, specific depletion of targeted Lmo2, Zbtb1, and Cbfa2t3 proteins. sgRNA against *Lmo2*, *Zbtb1*, *Cbfa2t2*, or *Cbfa2t3* was introduced into the Cas9-expressing (GFP^+^) LPs. Four days after sgRNA transduction, nuclear lysates from retrovirus infected GFP^+^hNGFR^+^ cells were subjected to immunoblotting for Lmo2, Zbtb1, Cbfa2t3, Bcl11a, and LaminB. Two independent experiments were performed with similar results. *B*, an experimental scheme for the deletion of *Lmo2*, *Zbtb1*, *Cbfa2t3*, and *Cbfa2t2* using the CRISPR/Cas9 system in *Ebf1*-deficient LPs is shown. *C*, retroviral vectors encoding sgRNAs against luciferase (sgControl), *Lmo2* (sgLmo2), *Zbtb1* (sgZbtb1), *Cbfa2t3* (sgCbfa2t3), or *Cbfa2t2* (sgCbfa2t2) were introduced into Cas9-expressing (GFP^+^) LPs. Five days after sgRNA introduction, LPs were transferred onto OP9-DLL4 stromal cells and cocultured for 2 days. GFP^+^hNGFR^+^ sgRNA transduced cells were gated and analyzed for CD44 and CD25 expression. *D*, the percentage of CD25^+^ cells among GFP^+^hNGFR^+^ sgRNA transduced cells (*C*) is shown with standard deviation (SD). *E*, ten days after sgRNA introduction, LPs were transferred onto OP9-DLL4 stromal cells and cocultured for 2 days. GFP^+^hNGFR^+^ sgRNA-transduced cells were gated and analyzed for CD44 and CD25 expression. *F*, the percentage of CD25^+^ cells among GFP^+^hNGFR^+^ sgRNA transduced cells (*E*) is shown with SD. Data are representative of two (*A*) or three (*C* and *E*) independent experiments. Data represent the mean values of three independent biological replicates (*D* and *F*). ∗∗*p* < 0.01 by two-sided Student’s *t* test. LP, lymphoid progenitor; sgRNA, single-guide RNA.

To verify whether Zbtb1 and Cbfa2t3 are involved in the maintenance of the T-lineage potential, we performed CRISPR/Cas9-mediated acute disruption of *Zbtb1* and *Cbfa2t3* in *Ebf1*-deficient LPs (22, 31). Cas9-GFP-transduced *Ebf1*-deficient LPs were infected with bicistronic retroviral vectors carrying single-guide RNAs (sgRNAs) against luciferase (control), *Lmo2*, *Zbtb1*, *Cbfa2t3*, or *Cbfa2t2* with human nerve growth factor receptor (hNGFR) marker. Specific losses of the targeted proteins were detected by immunoblotting in Cas9-expressing LPs, 4 days after sgRNA introduction (Fig. 2*A*). Five or ten days after sgRNA transduction, Cas9-GFP^+^sgRNA-hNGFR^+^ *Ebf1*-deficient LPs were provided Notch stimulation *via* transferring onto a OP9-DLL4 monolayer to examine their capacity to differentiate into the T-lineage (Fig. 2*B*). Notably, deletion of *Zbtb1* in *Ebf1*-deficient LPs slightly or significantly reduced cell recovery at 5 or 10 days post-sgRNA introduction (dpi), respectively, before Notch stimulation (Fig. S2, *A* and *B*). These results are agreed with previous reports showing the functional importance of Zbtb1 in preventing p53-mediated apoptosis in LPs (46, 47). Two days after DLL4-mediated Notch stimulation, the developmental status of sgRNA-transduced *Ebf1*-deficient LPs was scored using the markers, CD44 and CD25, which distinguish CD4^−^CD8^−^ (double-negative; DN) T progenitor stages (Fig. 2*B*, right) (2, 13). Deletion of *Zbtb1* severely impaired the progression of LPs into DN2 (CD44^+^CD25^+^) stage at 5 and 10 days after sgZbtb1 transduction. These results suggest that LPs sharply lost the T-lineage potential in 5 days after acute disruption of *Zbtb1*, whereas, in agreement with a previous report (31), deletion of *Lmo2* induced loss of ability to differentiate into T-lineage gradually, around 10 days (Figs. 2, *C*–*F* and S2, *C* and *D*). While *Cbfa2t2* disruption did not have a significant effect on the generation of CD25^+^ cells after Notch stimulation, *Cbfa2t3*-deficient LPs had modestly reduced DN2 cells at 5 dpi and were arrested at DN1 stage as well as *Lmo2*- or *Zbtb1*-deficient LPs at 10 dpi (Figs. 2, *C*–*F* and S2, *C* and *D*). Therefore, in addition to Lmo2, Zbtb1 and Cbfa2t3 would play a crucial role in the maintenance of T-lineage differentiation capacity triggered by Notch signaling in LPs with minor effects on the cell recovery (Fig. S2*A*), while expression levels of Notch1 and Notch2 in *Lmo2*-, *Zbtb1*-, or *Cbfa2t3*-deficient LPs were comparable with those of sgControl-transduced cells (Fig. S2*E*).

### Zbtb1 regulates *Tcf7* expression levels in LPs

**Figure 3:**
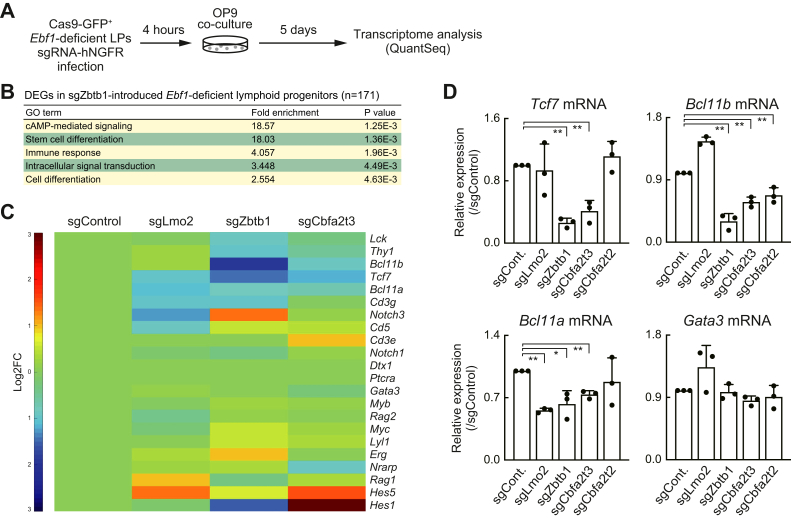
**Zbtb1 and Cbfa2t3 are involved in the regulation of *Tcf7* expression in LPs.***A*, experimental scheme for the transcriptome analysis is shown. Five days after sgRNA introduction, GFP^+^hNGFR^+^ LP cells were sorted, and subjected to QuantSeq 3′ mRNA sequencing. *B*, GO annotation was performed using the DAVID analysis tool. Top five GO terms for DEGs (*p* < 0.05, |log2FC| > 1, and TPM > 10) in sgZbtb1-introduced *Ebf1*-deficient LPs are shown. The full lists of the DEGs in *Lmo2*-, *Zbtb1*-, *Cbfa2t2*-, or *Cbfa2t3*-deficient LPs are shown in Table S2. Data are based on three independent biological replicates. *C*, heat map showing the expression changes of representative Notch-activated genes in pro-T cell stages (10) in response to the deletion of *Lmo2*, *Zbtb1*, or *Cbfa2t3*. Data are based on the average of three biological replicates. *D*, five days after sgRNA introduction, GFP^+^hNGFR^+^ LP cells were sorted (*A*). Relative expression levels of *Tcf7*, *Bcl11b*, *Bcl11a*, and *Gata3* against *Actb* were determined by RT-qPCR. The relative expression against sgControl-introduced cells is shown with SD. ∗∗*p* < 0.01, ∗*p* < 0.05 by two-sided Student’s *t* test. Data are based on three biological replicates. DEG, differentially expressed gene; GO, Gene Ontology; LP, lymphoid progenitor; sgRNA, single-guide RNA; TPM, transcripts per kilobase million.

To explore Zbtb1 and Cbfa2t3 target genes, which are involved in the regulation of T-lineage potential, Cas9-GFP^+^sgRNA-hNGFR^+^ LPs were sorted for transcriptome analysis (QuantSeq 3′ mRNA sequencing) at 5 days post sgRNA transduction, where the *Zbtb1* disruption had little impact on cell recovery (Fig. 3*A*). Differentially expressed genes (DEGs) affected by the disruption of *Lmo2*, *Zbtb1*, *Cbfa2t2*, and *Cbfa2t3* were defined by *p* value <0.05, |Log2 fold change| >1 and average transcripts per kilobase million (TPM) >10 in the control or samples. The number of DEGs in *Zbtb1* KO LPs, which had the most significant defect in T-lineage differentiation capacity among the samples, was 171, which was higher than that in *Lmo2*, *Cbfa2t2*, or *Cbfa2t3* KO cells (∼50 DEGs) (Table S2). Gene Ontology (GO) analysis showed that Zbtb1-regulated genes (171 DEGs in *Zbtb1* KO LPs) were enriched for genes categorized to “stem cell differentiation” and “cell differentiation” (Fig. 3*B*). We previously showed that *Tcf7* (encoding TCF1), the earliest Notch activated gene, is a functionally important Lmo2 target in LPs (31). Among Notch-dependent T-lineage signature genes in the earliest T progenitors (DN1-DN2a stages) (10), the expression levels of *Tcf7*, *Bcl11b*, and *Bcl11a* were decreased in *Zbtb1* or *Cbfa2t3* disrupted LP cells (Fig. 3*C*), while some deviations among replicates were observed because of their low expression levels in LPs without Notch signaling (TPM values for *Tcf7*; sgCont 3.78 ± 2.03 *versus* sgZbtb1 0.49 ± 0.01). We confirmed these observations *via* reverse transcription-quantitative PCR analysis. Among two of the earliest Notch target genes, *Tcf7* and *Gata3* (Fig. S1*B*), the expression levels of *Tcf7* were significantly decreased by the acute deletion of *Zbtb1* or *Cbfa2t3* but levels of *Gata3* were not affected (Fig. 3*D*). *Bcl11b*, which is activated at the later time point around the transition from DN2a to DN2b stages after Notch stimulation (Fig. S1*B*), was downregulated in not only *Zbtb1*- and *Cbfa2t3*-deficient but also *Cbfa2t2*-deficient LPs. The other Bcl11 family gene, *Bcl11a*, which is highly expressed in LPs (Fig. S1*B*) and has been reported to regulate the survival of LPs (48), had slightly lower mRNA and protein expression levels in the *Zbtb1* KO LPs (Figs. 2*A* and 3*D*). These data, along with a previous report (31), demonstrate that *Tcf7* appears to be a functionally important common target gene of Zbtb1, Cbfa2t3, and Lmo2 on the T-lineage differentiation capacity of LPs.

### Zbtb1 binds to the upstream regions of the *Tcf7* locus

**Figure 4:**
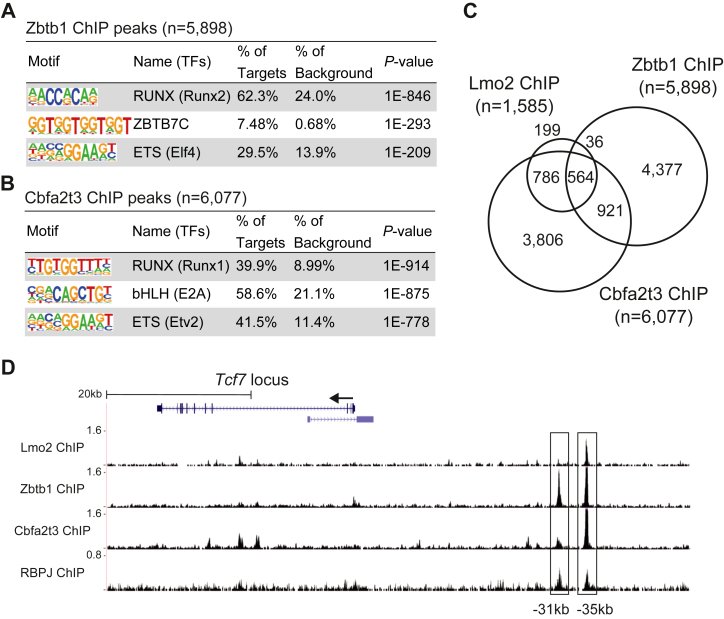
**Lmo2/Cbfa2t3/Zbtb1 complex binds to the −35 kb region of the *Tcf7* locus.***A*, chIP-seq analyses against Zbtb1 and Cbfa2t3 were performed using *Ebf1*-deficient LPs without Notch stimulation. The top three enriched sequence motifs among the 5898 reproducible Zbtb1 peaks are shown. *B*, top three enriched sequence of motifs among the 6077 reproducible Cbfa2t3 peaks are shown. *C*, Venn diagrams show the numbers of reproducible Zbtb1 and Cbfa2t3 ChIP peaks with the previously reported Lmo2 peaks (31) in *Ebf1*-deficient LPs. *D*, representative ChIP-seq tracks for Lmo2, Zbtb1, and Cbfa2t3 in *Ebf1*-deficient LPs without Notch stimulation, and RBPJ in DN1 cells (10) around the *Tcf7* locus are shown. The −31 and −35 kb RBPJ-binding sites are labeled with rectangles. Data are based on ChIP-seq peaks scored as reproducible in two replicate samples (*A*–*C*), or representative of two independent experiments (*D*). ChIP-seq, chromatin immunoprecipitation followed by deep sequencing; DN, double-negative; LP, lymphoid progenitor.

The Zbtb1-sensitive genes are regulated both directly and indirectly. Thus, we performed chromatin immunoprecipitation followed by deep sequencing (ChIP-seq) for Zbtb1 and Cbfa2t3 in *Ebf1*-deficient LP cells and compared them with the Lmo2 occupancy genomic regions (31). Approximately 6000 of reproducible Zbtb1 and Cbfa2t3 binding peaks were detected across the genome, and the most enriched sequence was the Runx motif, which has been found around hematopoietic transcription factors, including Lmo2, E2A, PU.1, Bcl11b, or GATA3, occupancy sites (Fig. 4, *A* and *B*) (24, 31, 49, 50). The second enriched transcription factor–binding motifs were the Zbtb7c binding motif for Zbtb1 and the bHLH motif, which is also enriched around the Lmo2 binding regions (31), for Cbfa2t3 (Fig. 4, *A* and *B*). Indeed, more than 35% and 85% of the Lmo2 binding sites were co-occupied with Zbtb1 and Cbfa2t3, respectively, and 564 peaks were cobound by all three molecules (Fig. 4*C*). The expression levels of *Bcl11a* were moderately regulated by Lmo2 and Cbfa2t3 (Fig. 3*D*), and these two factors co-occupied the downstream regions of the *Bcl11a* locus without Zbtb1 binding (Fig. S3, rectangles). One of the representative sites cobound by Lmo2, Zbtb1, and Cbfa2t3 was the −35 kb upstream region of the *Tcf7* locus, which overlapped with one of the RBPJ (also known as CSL), a DNA-binding subunit of the intracellular domain of Notch (Notch-IC) complex, binding sites after Notch stimulation (Fig. 4*D*, right rectangle) (10). We have previously reported that Lmo2 directly binds to the −35 kb upstream region of the *Tcf7* locus and maintains DNA methylation status of the *Tcf7* locus (31). Therefore, the Lmo2/Zbtb1/Cbfa2t3 complex appears to regulate the expression of *Tcf7 via* direct binding at the −35 kb region of the *Tcf7* locus in LPs before the progenitor cells receive Notch signal. Importantly, a clear Zbtb1 peak and a modest Cbfa2t3 peak with almost no Lmo2 binding signal were detected at the other RBPJ-binding site in the −31 kb upstream region of the *Tcf7* locus, which has been reported as a Notch-dependent enhancer region of *Tcf7* (Fig. 4*D*, left rectangle) (15, 51). Thus, Zbtb1 may be involved in Notch-mediated activation of *Tcf7* in Lmo2-dependent and Lmo2-independent mechanisms.

### Defect in the T-lineage potential of *Zbtb1*-deficient LPs is recovered *via Tcf7*-transduction

**Figure 5:**
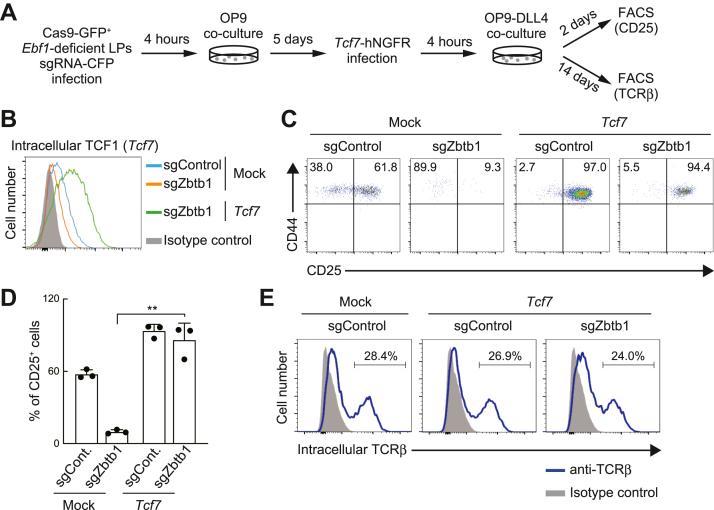
**Introduction of *Tcf7* rescues the generation of CD25**^**+**^**cells in *Zbtb1*-deficient LPs.***A*, experimental scheme for the transduction of *Tcf7* after the deletion of *Zbtb1* in *Ebf1*-deficient LPs is shown. *B*, two days after *Tcf7* (encoding TCF1 protein) transduction (*A*), GFP^+^CFP^+^hNGFR^+^ cells were gated and analyzed for intracellular TCF1 expression. Data are representative of two independent experiments. *C*, Cas9-GFP and sgRNA-CFP-transduced LPs were introduced with *Tcf7*-hNGFR, and transferred onto OP9-DLL4 stromal cells for 2 days. GFP^+^CFP^+^hNGFR^+^ cells were gated and analyzed for CD44 and CD25 expression. Data are representative of three independent experiments. *D*, the percentage of CD25^+^ cells among GFP^+^CFP^+^hNGFR^+^ cells (*C*) is shown with SD. The data represent the mean values of three independent biological replicates. ∗∗*p* < 0.01 by two-sided Student’s *t* test. *E*, fourteen days after Notch stimulation (*A*), GFP^+^CFP^+^hNGFR^+^ cells were gated and analyzed for intracellular TCRβ expression. Data are representative of two independent experiments. sgRNA, single-guide RNA.

Next, to test whether insufficient expression of *Tcf7* led to the loss of T-lineage potential in *Zbtb1*-deficient LPs, we examined the effects of *Tcf7* introduction in *Zbtb1* KO LPs. Five days after the introduction of sgZbtb1, marked with a CFP reporter, LP cells were transduced with a second retrovirus encoding *Tcf7*-hNGFR and then cocultured with OP9-DLL4 to initiate T-lineage differentiation for 2 days (Fig. 5*A*). Transduction of *Tcf7* dramatically restored the generation of CD44^+^CD25^+^ DN2 cells in *Zbtb1*-deficient LPs, while a partial rescue of cell recovery was observed after *Tcf7* introduction (Figs. 5, *B*–*D* and S4*A*). Moreover, *Tcf7*-introduced *Zbtb1*-deficient LPs clearly expressed the intracellular TCRβ chain, 14 days after Notch stimulation (Fig. 5*E*). Consequently, these results suggest that *Tcf7* is a major downstream target of Zbtb1 for the maintenance of the T-lineage capacity of LPs and that other mechanisms contribute to the regulation of Zbtb1-mediated proliferation and/or survival of LP cells.

### Zbtb1 regulates the ability of primary BM progenitors to differentiate into T-lineage

**Figure 6:**
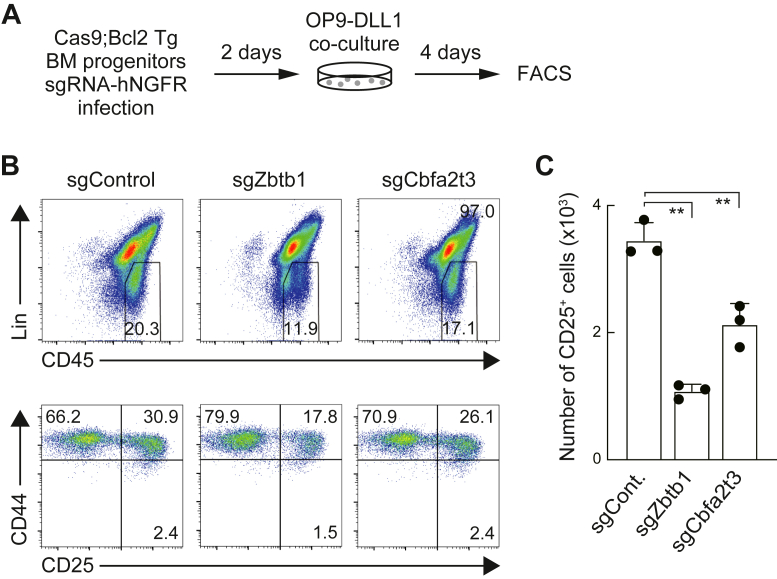
***Zbtb1*-deficient BM progenitors have attenuated ability to differentiate into T-lineage.***A*, experimental scheme for the deletion of *Zbtb1* and *Cbfa2t3* using the CRISPR/Cas9 system in BM progenitor cells is shown. BM progenitors obtained from Cas9;Bcl2 Tg mice were transduced with sgRNA against *Zbtb1* and *Cbfa2t3* and cultured without stroma cells for 2 days. Then, they were transferred onto OP9-DLL1 and cocultured for 4 days. hNGFR^+^ sgRNA-transduced cells were gated and analyzed for Lin markers, CD45, CD44, and CD25 expression. *B*, flow cytometric analysis of sgRNA-transduced BM progenitors cocultured with OP9-DLL1 for 4 days. Representative Lin/CD45 profiles in hNGFR^+^ sgRNA-introduced cells and CD44/CD25 profiles in hNGFR^+^Lin^−^CD45^high^ cells are shown. The results are representative of three independent experiments. *C*, the number of CD25+ cells among hNGFR+ sgRNA transduced cells (*B*) is shown with SD. The data represent the mean values of three independent biological replicates. ∗∗*p* < 0.01 by two-sided Student’s *t* test. BM, bone marrow; sgRNA, single-guide RNA.

Finally, we verified the effects of *Zbtb1* disruption on the developmental potential of the T-lineage in primary cells. BM-derived hematopoietic progenitor cells from Rosa26-Cas9 knock-in mice with a Bcl2 transgene (Cas9;Bcl2 Tg), which enhances viable recovery of cells without altering T-cell development (52), were transduced with sgRNA against *Zbtb1* or *Cbfa2t3* and cultured without OP9 stromal cells for 2 days. The cells were then transferred onto an OP9-DLL1 monolayer to induce Notch signaling (Fig. 6*A*). Four days after Notch stimulation, the progression of early T-cell development was assessed using lineage markers (Lin), CD45, CD44, and CD25 expression. The percentages of Lin^−^CD45^high^ lymphoid cells (Fig. 6*B*, upper) and CD44^+^CD25^+^ cells (Fig. 6*B*, lower) were moderately reduced by disruption of *Zbtb1* and *Cbfa2t3*, and the recovery of Lin^−^CD45^high^CD44^+^CD25^+^ DN2 cells in the cultures was significantly attenuated (Fig. 6, *B* and *C*). These results demonstrated that Zbtb1 and Cbfa2t3 regulate the capacity of primary hematopoietic progenitor cells to differentiate into T-lineage cells.

## Discussion

Biochemical analyses of Lmo2 protein complexes in blood tumor cells revealed that Lmo2 organizes large transcriptional complexes with bHLH and GATA family members. However, the subunits of Lmo2 complexes in physiological HPCs have remained obscure until now. We recently revealed that Lmo2 is required to maintain the ability of LPs to differentiate into T-lineage (31). Thus, in this study, we attempted to identify the functional binding partners of Lmo2, which are essential for the maintenance of T-lineage potential, in LPs using a biochemical approach. To purify the Lmo2 protein complexes, we employed an *Ebf1*-deficient LP cell line, which retained the ability to differentiate into T-lineage in a Notch signaling–dependent manner (31). In addition to the previously reported Lmo2-binding molecules in blood tumors, including Cbfa2t3, the transcription factor, Zbtb1, has been identified as a novel interacting partner of Lmo2 in LPs.

The functional importance of Zbtb1 in lymphopoiesis has been reported in two independent mouse models. The first model was established using a chemically induced mouse mutation system. A missense mutation of the conserved cysteine to arginine (C74R) in the BTB domain of Zbtb1 causes cell-intrinsic defects in lymphopoiesis, especially in the earliest stage of T-cell development; thus, this mouse model was named *scanT* (42). In another model, *Zbtb1* was unexpectedly found as an insertion site of a transgene, which led to the T^−^B^+^NK^−^ severe combined immunodeficiency phenotype. A similar severe combined immunodeficiency phenotype has been observed in the germline disruption of *Zbtb1* (43). Therefore, the loss of function of Zbtb1 clearly induces a disorder of lymphopoiesis, particularly in T-cell development (42, 43). However, the molecular mechanisms underlying how Zbtb1 controls the initiation of T-cell program have not been clarified. In this study, we showed that Zbtb1 directly binds to the *Tcf7* locus, which is activated by the Notch-IC/RBPJ transcriptional complex to trigger T-cell development, as a component of the Lmo2 complex, and maintains the ability of LPs to differentiate into T-lineage. *Zbtb1* deficiency had a much more striking effect than *Cbfa2t3* or *Lmo2* disruption in maintaining the T-lineage potential, especially 5 days after sgRNA transduction (Fig. 2). Lmo2 and Cbfa2t3 are non-DNA–binding components of the transcriptional complexes (32, 33, 45). In contrast, ZBTB family members are known to bind directly to specific DNA sequences *via* C-terminal zinc finger domains (53). The Zbtb1-binding sites detected by ChIP-seq analysis in LPs had a significantly enriched consensus motif for a ZBTB family member (Fig. 4*A*). Indeed, there is a 5′-TGGTGGT-3′ motif at the center of the −35 kb upstream region of the *Tcf7* transcriptional start site. Thus, Zbtb1 would act as a part of the DNA-binding subunits of the Lmo2 complex at the *Tcf7* locus. This may be one of the reasons why *Zbtb1* disruption quickly induced the loss of the ability to differentiate into T-lineage in LPs.

*Zbtb1* disruption leads not only to defects in T-cell development but also to lower cell recovery (Fig. S2, *A* and *B*). Bcl11a has been reported as an essential transcription factor for the survival and proliferation of LPs *via* the activation of negative regulators of p53 activities, including Bcl2, Bcl-xL, and Mdm2 (48). In fact, expression levels of Bcl2 were modestly decreased in *Zbtb1*-deficient LPs (sgCont 678.5 TPM *versus* sgZbtb1 455.0 TPM, *p* < 0.01) (GSE199696). Therefore, the lower expression levels of *Bcl11a* in *Zbtb1*-deficient LPs (Figs. 2*A* and 3*D*) may contribute to this lower cell recovery. As there was no significant Zbtb1-binding signal around the *Bcl11a* locus (Fig. S3), Zbtb1 may regulate *Bcl11a* expression indirectly *via* the regulation of activators or repressors of *Bcl11a* or directly mediated by unknown distal *cis*-regulatory elements for *Bcl11a*. Zbtb1 has also been reported to be involved in maintaining genomic integrity. Zbtb1 regulates the recruitment of phosphorylated KAP-1 and RAD18 to DNA damage sites and subsequent DNA synthesis using error prone DNA polymerase to avoid cell death during DNA replication across damaged DNA (46). In addition, actively proliferating LPs from *ScanT* mice have a significantly higher frequency of p53-mediated apoptosis (47). Consequently, Zbtb1 may regulate the survival and proliferation of LPs *via* several distinct mechanisms.

In this study, we also examined the roles of the transcriptional corepressor Cbfa2t3, a previously reported Lmo2-interacting molecule in B-cell lymphoma and the erythrocytic lineage (40, 41). As shown here, approximately 85% of Lmo2 binding genomic regions were cobound by Cbfa2t3 in LPs (Fig. 4*C*); thus, these factors closely cooperate to regulate gene expression across the genome. A significant defect in T-cell development has been observed in *in vivo* and *in vitro* experimental systems using BM progenitors from *Cbfa2t3*-deficient mice (44, 45). Cbfa2t3 is not only involved in T lymphopoiesis but is also known to regulate the proper integration of Notch signals by interactions with Notch-IC and RBPJ. When Notch receptors interact with Notch ligands, Notch-IC is cleaved and translocated into the nucleus. Notch-IC organizes transcriptional complexes with RBPJ and activates its target genes (8). Cbfa2t3 interacts with RBPJ, prebinds to Notch target loci, and appears to regulate the repression and activation of Notch target genes, before and after Notch stimulation, respectively (44). This model fits to the role of the Lmo2/Cbfa2t3/Zbtb1 complex, which was found in this and previous reports, at the *Tcf7* locus (31). Lmo2, Zbtb1, and Cbfa2t3 cobound to the −35 kb region of the *Tcf7* locus in LP cells before Notch stimulation and regulated *Tcf7* expression activated by the Notch-IC/RBPJ complex when Notch stimulation was provided. Importantly, while re-expression of intact *Cbfa2t3* into *Cbfa2t3* disrupted hematopoietic progenitors restores the T-lineage potential, *Cbfa2t3*-deficient progenitors with the *Cbfa2t3* mutant, which has a truncated Notch-IC binding domain, failed to differentiate into T-cells (44). Thus, prebinding of Lmo2 complexes, including Cbfa2t3, to Notch target loci in LPs would have a crucial role in the appropriate activation of Notch target genes, including *Tcf7*, and in triggering the T-cell differentiation program in the thymus.

TCF1, encoded by *Tcf7*, is an essential transcription factor in the earliest stages of T-cell development (54). Together with Runx transcription factors, Notch signaling is required to activate *Tcf7* expression in T-cell progenitors (10, 12, 15, 22). There are two Notch-IC/RBPJ and Runx1 binding sites upstream of the *Tcf7* locus at −31 kb and −35 kb regions (10, 12). Among these, the −31 kb region appears to be more important than the −35 kb region as a Notch-dependent enhancer of *Tcf7* (15, 51). Although the physiological importance of the −35 kb region, a Lmo2/Cbfa2t3/Zbtb1 complex binding site, has not been clarified, our previous data suggest that Lmo2 maintains the transcriptionally poised chromatin state of the *Tcf7* locus by direct binding to the −35 kb region in LPs (31, 51). Therefore, organization of the Lmo2/Cbfa2t3/Zbtb1 complex in the −35 kb region would play an important role in maintaining the accessible chromatin configuration of the *Tcf7* locus and the ability to differentiate into T-lineage. In addition to the −35 kb region, Zbtb1 binds to the −31 kb region of the *Tcf7* locus without cobinding of Lmo2 (Fig. 4*D*) (51). Thus, Zbtb1 would be involved in the regulation of *Tcf7* expression in Lmo2-dependent and Lmo2-independent manners in the −35 kb and −31 kb regions, respectively. Only 10% of Zbtb1 binding genomic regions were co-occupied with Lmo2 (Fig. 4*C*). In addition, the expression of Zbtb1 is maintained at high levels not only during T-cell development in the thymus but also in mature T cells in the periphery, while Lmo2 and Cbfa2t3 expression are sharply downregulated after the earliest pro-T cell stages in the thymus (Fig. S1*B*) (34). Therefore, the roles of Zbtb1 in T-cell development and function are thought to be beyond those of the Lmo2 complexes. Additionally, the importance of Zbtb1 in the generation of group 3 innate lymphoid cells (ILCs) has been reported (55). The lineage determination of ILCs from LPs is controlled by *Tcf7* (56), and the initiation of *Tcf7* expression is regulated by the −31 kb upstream regulatory element of the *Tcf7* locus (51), where a strong Zbtb1-binding signal was observed in LPs (Fig. 4*D*). Thus, in addition to T-cell development, the Zbtb1-Tcf7 axis may contribute to the development of ILCs.

Here, we identified Zbtb1 as a novel functional subunit of the Lmo2 complex, using proteomic analysis and an acute deletion system in *Ebf1*-deficient LP cell lines. Moreover, the functional importance of Zbtb1 and Cbfa2t3 was confirmed in primary BM progenitors. Our results indicate that the T-lineage potential of LPs is actively maintained by the cooperative effects of various transcription factors, including Lmo2, Cbfa2t3, and Zbtb1 (Fig. S4*B*).

## Experimental procedures

### Mice

*Ebf1*-deficient mice were kindly provided by Dr Rudolf Grosschedl (Max Planck Institute of Immunobiology and Epigenetics) (57). B6.Cg-Tg(BCL2)25Wehi/J (Bcl2 Tg) (58) and B6.Gt(ROSA)26^Sortm1.1(CAG-cas9^∗^,-EGFP)Fezh^/J (Rosa26-Cas9 knock-in) (59) mice were purchased from the Jackson Laboratory. All animals were bred and maintained in the animal facility of Tokai University School of Medicine, under specific pathogen-free conditions, and the protocol supporting animal breeding for this work was reviewed and approved by the Animal Experimentation Committee of Tokai University.

### Cell culture of *Ebf1*-deficient lymphoid progenitor lines

*Ebf1*-deficient lymphoid progenitor cell lines (31) were cultured in IMDM (Wako) with 10% fetal bovine serum (Sigma–Aldrich), penicillin–streptomycin–glutamine, 50 μM β-mercaptoethanol (Sigma–Aldrich), 10 ng/ml mouse SCF (PeproTech), 10 ng/ml human Flt3L (PeproTech), 10 ng/ml mouse IL-7 (PeproTech) with mitomycin C (Wako) treated OP9. For T-cell induction, lymphoid progenitors were cocultured on OP9-DLL4 for 2 days under the same conditions as the maintenance of the lymphoid progenitors.

### Flow cytometry

For staining of sgRNA introduced LP cells, surface antibodies against Notch1 PE (BioLegend; 130607), Notch2 PE (BioLegend; 130707), CD44 PECy7 (BioLegend; 103029), CD25 APC-e780 (eBioscience; 47-0251-82) and human-NGFR PE (BioLegend; 345106) were used. For intracellular staining, antibodies against TCF1 PE (CST; 14456) with transcription factor buffer set (BD; 562574) and TCRβ PE (BioLegend; 109208) with Cyto-Fast Fix/Perm Buffer Set (BioLegend; 426803) were used.

All of the cells were analyzed using a flow cytometer, FACSVerse (BD), FACSLyric (BD), FACSAria Fusion (BD), or LSRFortessa (BD) with FlowJo software (Tree Star).

### Cell culture of primary BM progenitors

BM was removed from the femurs of 3- to 4-month-old Rosa26-Cas9 knock-in mice with a Bcl2 transgene (Cas9;Bcl2 Tg). Suspensions of BM cells were stained for lineage (Lin) markers using biotin-conjugated lineage antibodies (CD11b) ([BioLegend; 101204], CD11c [BioLegend; 117304], Gr-1 [BioLegend; 108404], TER-119 [BioLegend; 116204], NK1.1 [BioLegend; 108704], CD19 [BioLegend; 115504], and CD3ε [BioLegend; 100304]), then incubated with antibiotin magnetic beads (Miltenyi Biotec), and passed through a magnetic column using AutoMACS with the ‘Deplete’ program (Miltenyi Biotec). The hematopoietic progenitors were infected with retroviral vectors encoding sgRNA and cultured using the OP9 medium (α-MEM (Sigma), 20% fetal bovine serum, 50 μM β-mercaptoethanol, Pen-Strep-Glutamine) supplemented with 10 ng/ml of human IL-7, 10 ng/ml of mouse SCF, and 10 ng/ml of human Flt3L for 2 days, then transferred onto OP9-DLL1 and cocultured for 4 days (60). The cultured cells were then disaggregated, filtered through a 40 μm nylon mesh, and subjected to flow cytometry analysis using surface antibodies against CD45 PECy7 (BioLegend; 103113), CD44 FITC (BioLegend; 103005), CD25 APC-e780, human-NGFR PE, and a biotin-conjugated lineage cocktail (CD8α, CD11b, CD11c, Gr-1, TER-119, NK1.1, CD19, TCRβ (BioLegend; 109204), and TCRγδ (BioLegend; 118103)) with streptavidin PerCPCy5.5 (BioLegend; 405214). Prior to cell surface staining, cells were treated with Fc blocker (Miltenyi).

### Two-step affinity purification of Lmo2 complexes from lymphoid progenitors

Lymphoid progenitor cells were infected with either mock control (pMxs-IRES-hNGFR) or Myc-Flag-Lmo2-containing retrovirus. Three days after infection, Myc-Flag-tagged Lmo2-infected hNGFR^+^ cells were solubilized with the following protease inhibitor-containing immunoprecipitation buffer: 50 mM Tris–HCl (pH 7.5), 150 mM NaCl, 10% glycerol, 0.1% Tween, 1 mM EDTA, 10 mM NaF, 1 mM DTT, and a protease inhibitor cocktail (Roche Applied Science) and lysed on ice for 30 min with gentle shaking and sonicated on a VP-55 sonicator (TAITEC) for three cycles, amplitude 6 for 20 s, followed by 1 min rest. The insoluble materials were removed by centrifugation, and immunoprecipitation with mouse control Ig agarose (Sigma–Aldrich, A0919) or anti-Flag M2 agarose (Sigma–Aldrich, A2220) was performed overnight at 4 °C. Immune complexes were eluted from the agarose by 3×Flag peptide (Sigma–Aldrich), and the eluted Lmo2 complexes were subjected to a second immunoprecipitation with anti-Myc magnetic beads (MBL). Immune complexes were eluted from the magnetic beads with Myc peptide (MBL) and separated by SDS-PAGE. The bands were excised from the gel and subjected to a mass spectrometric analysis to identify corresponding proteins. The gel pieces were washed twice with 100 mM bicarbonate in acetonitrile, and the proteins were digested with trypsin. After adding 0.1% formic acid to the supernatant, the peptides were analyzed by LC-MS/MS with an Advance ultrahigh performance liquid chromatograph (Bruker) and an Orbitrap Velos Pro mass spectrometer (Thermo Fisher Scientific). The resulting tandem mass spectrometry dataset was analyzed using the Mascot software program (Matrix Science). Mascot score is the probability that the observed match is a random event (Mascot score > 100 means the absolute probability < 1e-10). GO analysis was performed using the DAVID analysis tool (https://david.ncifcrf.gov).

### Cloning

Human *TCF7* complementary DNA was inserted into a multicloning site of the pMxs-IRES-hNGFR vector. sgRNA expression vector (E42-dTet) and Cas9-GFP expression vector were described previously (22). 20-mer sgRNAs were designed using the Benchling web tool (https://www.benchling.com) and inserted into the empty sgRNA expression vector by PCR-based insertion. Two sgRNA expression vectors were generated for one gene, and pooled retroviral plasmids were used to make retroviral supernatant. Sequences of sgRNAs used in this study are listed below.

sgControl (Luciferase) #1; 5′-accgcgaaaaagttgcgcgg-3′

sgControl (Luciferase) #2; 5′-ggcatgcgagaatctcacgc-3′

sgLmo2 #1; 5′-gcggtgactgtccttgagcg-3′

sgLmo2 #2; 5′-cagcggagcgaccgagcaag-3′

sgZbtb1 #1; 5′-ctgctcgaaactggaaggag-3′

sgZbtb1 #2; 5′-agctcaacaaccaaagagag-3′

sgCbfa2t2 #1; 5′-caataaatcctggaggaccg-3′

sgCbfa2t2 #2; 5′-cgttactgctgacgatgtgg-3′

sgCbfa2t3 #1; 5′-ctgcgtcttcacttcagccg-3′

sgCbfa2t3 #2; 5′-tgggtgtagatggggacc-3′

### CRISPR/Cas9-mediated deletion of target genes in lymphoid progenitors

Lymphoid progenitor cells were transduced with retroviral vectors encoding Cas9-GFP, and 3 days after infection, GFP^+^ retrovirus-infected cells were sorted. Then, they were expanded for a week and subjected to the second retrovirus transduction with sgRNA-hNGFR or sgRNA-CFP. They were transferred onto OP9-DLL4 on day 5 or day 10 after second infection, then CD25 and CD44 profiles on Cas9^+^sgRNA^+^ retrovirus-infected cells were analyzed.

### Immunoblotting

Nuclear extracts were prepared using NE-PER Nuclear and Cytoplasmic Extraction Reagents (Pierce). Lysates were run on 10% polyacrylamide gel, followed by immunoblotting. The antibodies used for the immunoblot analysis were anti-LaminB (CST, 13435), anti-Myc (MBL; M192-3), anti-Zbtb1 (Bethyl; S303-242A), anti-Lmo2 (Novus, NB110-78626), anti-Bcl11a (CST; 75432), and anti-Cbfa2t3 (ProteinTech; 17190-1-AP).

### RNA preparation and RT-qPCR

Total RNA was isolated from samples of 3 × 10^5^ cultured cells using a RNeasy Micro Kit (Qiagen) according to the manufacturer’s instructions. Complementary DNA was synthesized with Super Script IV VILO (Thermo Fisher Scientific). Quantitative PCR was performed using Fast SYBR Green Master Mix (Thermo Fisher Scientific) and QuantStudio 3 (Applied Biosystems). The primer sets used in this study are listed below.

*Actb* FW; 5′-tacagcccggggagcat-3′

*Actb* RV; 5′-acacccgccaccagttc-3′

*Tcf7* FW; 5′-tgatgctgggatctggtgta-3′

*Tcf7* RV; 5′-cttgggttctgcctgtgttt-3′

*Bcl11b* FW; 5′-tggatgccagtgtgagttgt-3′

*Bcl11b* RV; 5′-gctgcttgcatgttgtgc-3′

*Gata3* FW; 5′-cttatcaagcccaagcgaag-3′

*Gata3* RV; 5′-cccattagcgttcctcctc-3′

*Bcl11a* FW; 5′-gcacttaagcaaacgggaat-3′

*Bcl11a* RV; 5′-caggtgagaaggtcgtggtc-3′

### QuantSeq 3′ mRNA sequencing

Total RNA was isolated from samples of 3 × 10^5^ cultured cells using a RNeasy Micro Kit (Qiagen). Five hundred nanograms of total RNA was subjected to the 3′mRNA library preparation with QuantSeq 3′ mRNA-Seq Library Prep Kit FWD (LEXOGEN) according to the manufacturer’s instructions. After the PCR step, size distribution and yield of the library was determined by the D1000 high sensitivity tape station (Agilent) or Agilent High Sensitivity DNA kit on the bioanalyzer (Agilent). The pooled libraries were loaded on the Illumina Nextseq500 platform and analyzed by 75 bp single read. Adapter sequences were trimmed from the raw RNA-seq reads with fastp. Trimmed reads of each sample were mapped to the reference mouse genome mm10 using STAR and normalized to one million reads in the original library. DEGs were defined with *p* < 0.05, |log2FC| > 1, and TPM > 10 based on measurements from three biologically independent replicates for each sample type. GO analysis was performed using the DAVID analysis tool (https://david.ncifcrf.gov).

### ChIP-seq

About 1 × 10^7^ *Ebf1*-deficient LPs without Notch stimulation were fixed with 1 mg/ml disuccinimidyl glutarate (Thermo Scientific) in PBS for 30 min at room temperature followed by an additional 10 min with addition of formaldehyde up to 1%. The reaction was quenched by addition of 1/10 volume of 0.125 M glycine and the cells were washed with Hanks' balanced salt solution (Gibco). Pelleted nuclei were dissolved in lysis buffer (0.5% SDS, 10 mM EDTA, 0.5 mM EGTA, 50 mM Tris–HCl (pH 8) and PIC) and sonicated on a Bioruptor (Diagenode) for 18 cycles of 30 s sonication followed by 30 s rest, with high power. Five micrograms of anti-Zbtb1 Abs (a mixture of 2.5 μg of S303-242A (Bethyl) and 2.5 μg of ab79455 (Abcam)) or anti-Cbfa2t3 (a mixture of 2.5 μg of 17190-1-AP (Proteintech) and 2.5 μg of sc-373691 (Santa Cruz)) were prebound to Dynabeads anti-rabbit Ig or protein A/G (Invitrogen) and then added to the diluted chromatin complexes. The samples were incubated overnight at 4 °C, then washed and eluted for 6 h at 65 °C in ChIP elution buffer (20 mM Tris–HCl, pH 7.5, 5 mM EDTA, 50 mM NaCl, 1% SDS, and 50 μg/ml proteinase K). Eluted chromatin fragments were cleaned up using ChIP DNA Clean & Concentrator (Zymo). ChIP-seq libraries were constructed using NEBNext Ultra II DNA Library Prep with Sample Purification Beads (E7103S, NEB) and NEBNext Multiplex Oligos for Illumina (E7500S, NEB) and sequenced on Illumina NextSeq500 in single read mode with the read length of 75 nt. Base calls were performed with RTA 1.13.48.0 followed by conversion to FASTQ with bcl2fastq 1.8.4 and produced approximately 30 million reads per sample. ChIP-seq data were mapped to the mouse genome build NCBI37/mm10 using Bowtie (v1.1.1; http://bowtie-bio.sourceforge.net/index.shtml) with “-v 3 -k 11 -m 10 -t --best –strata” settings and HOMER tagdirectories were created with makeTagDirectory and visualized in the UCSC genome browser (http://genome.ucsc.edu). ChIP peaks were identified with findPeaks.pl against a matched control sample using the settings “-P .1 -LP .1 -poisson .1 -style factor”. The identified peaks were annotated to genes with the annotatePeaks.pl command against the mm10 genomic build in the HOMER package. Peak reproducibility was determined by a HOMER adaptation of the IDR (Irreproducibility Discovery Rate) package according to ENCODE guidelines (https://sites.google.com/site/anshulkundaje/projects/idr). Only reproducible high quality peaks, with a normalized peak score ≥15, were considered for further analysis. Motif enrichment analysis was performed with the findMotifsGenome.pl command in the HOMER package using a 200 bp window.

### Statistical analysis

The statistical significance of differences between datasets was determined using two-sided Student’s *t* test using Excel. Statistical details of experiments can be found in the figure legends. In all figures, error bars indicate SD.

## Data availability

The accession number for all the new deep-sequencing data reported in this paper is GEO: GSE199696.

## Supporting information

This article contains supporting information.

## Conflict of interest

The authors declare that they have no conflicts of interest with the contents of this article.
