# Supplementary material for: Transcription factor Zbtb1 interacts with bridging factor Lmo2 and maintains the T-lineage differentiation capacity of lymphoid progenitor cells
Source: J Biol Chem. 2022 Sep 17;298(11):102506. doi: 10.1016/j.jbc.2022.102506 (PMC9582733; doi:10.1016/j.jbc.2022.102506)
Supplement: Supplemental Figures S1–S4 Legends [file mmc1.docx]

**Supporting information**

**Transcription factor Zbtb1 interacts with bridging factor Lmo2 and maintains the T-lineage differentiation capacity of lymphoid progenitor cells**

**Maria Koizumi, Yuichi Kama, Ken-ichi Hirano, Yusuke Endo, Tomoaki Tanaka, Katsuto Hozumi and Hiroyuki Hosokawa**

**Supplemental figure legends**

**Supplemental Figure S1**

(A), Full-size uncropped images of immunoblotting results in Fig. 1D are shown.

(B), RPKM values for *Lmo2*, *Zbtb1*, *Cbfa2t3*, *Tcf7*, *Gata3*, *Bcl11b*, and *Bcl11a* in common lymphoid progenitor (CLP) and DN subsets are shown (https://www.immgen.org; GSE100738) ([34](#_ENREF_34)).

**Supplemental Figure S2**

(A, B), The percentage of GFP^+^hNGFR^+^ sgRNA-transduced cells on five days (A) or ten days (B) after sgRNA transduction, before Notch stimulation, is shown with SD. Data represent the mean values of three independent biological replicates. ^**^*P* < 0.01 by two-sided Student’s *t*-test

(C, D), The number of CD25^+^ cells in Fig. 2C (C) or Fig. 2E (D) is shown with SD. The data represent the mean values of three independent biological replicates. ^**^*P* < 0.01 by two-sided Student’s *t*-test.

(E), Flow cytometric analysis of LPs, five days after sgRNA introduction, was performed (Fig. 2B). Representative Notch1 and Notch2 profiles in GFP^+^hNGFR^+^ sgRNA-introduced cells are shown. Three independent experiments were performed with similar results.

**Supplemental Figure S3**

Representative ChIP-seq tracks for Lmo2, Zbtb1, and Cbfa2t3 in LPs, and RBPJ in DN1 cells ([10](#_ENREF_10)) around the *Bcl11a* locus are shown. The Lmo2/Cbfa2t3 occupancy sites are labeled with rectangles. Data are representative of two independent experiments.

**Supplemental Figure S4**

(A), The number of GFP^+^CFP^+^hNGFR^+^CD25^+^ cells in Fig. 5C is shown with SD. Data represent the mean values of three independent biological replicates. ^**^*P* < 0.01 by two-sided Student’s *t*-test.

(B), A graphical summary of this study is shown. In lymphoid progenitors, Zbtb1 has a crucial role in maintaining the accessible chromatin configuration of the *Tcf7* locus and the ability to differentiate into T-lineage.
