# Supplementary figures and images for: Transcription factor Zbtb1 interacts with bridging factor Lmo2 and maintains the T-lineage differentiation capacity of lymphoid progenitor cells
Source: J Biol Chem. 2022 Sep 17;298(11):102506. doi: 10.1016/j.jbc.2022.102506 (PMC9582733; doi:10.1016/j.jbc.2022.102506)

Supplemental Figure S1

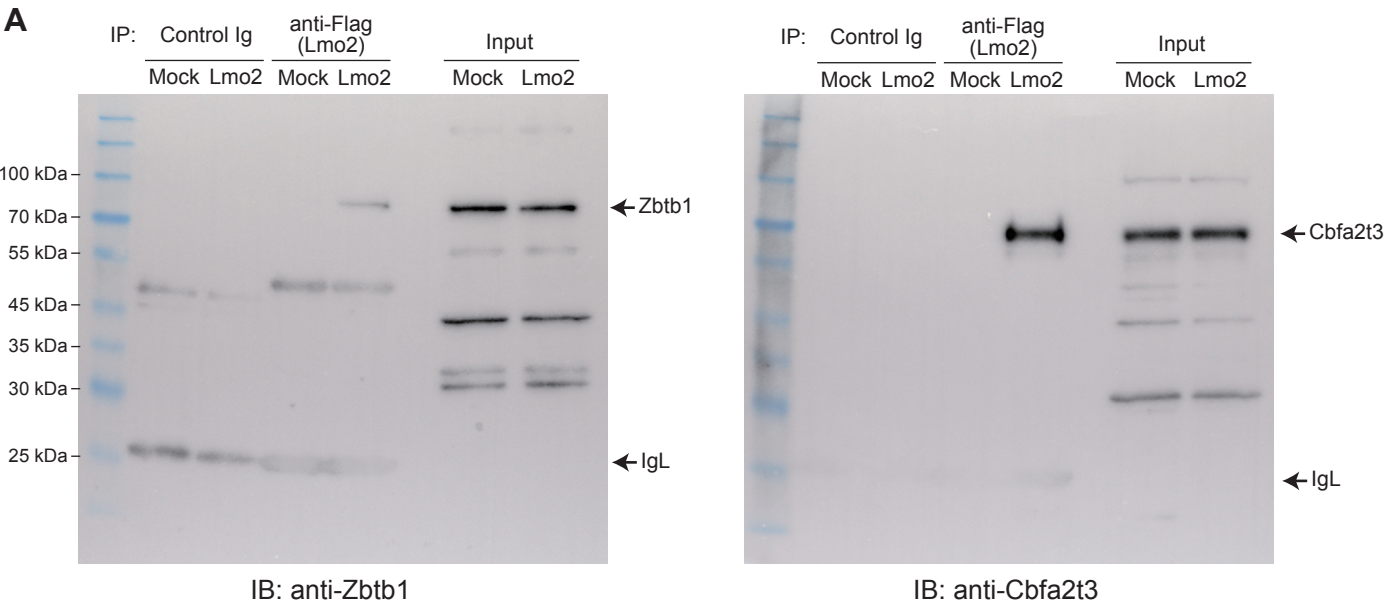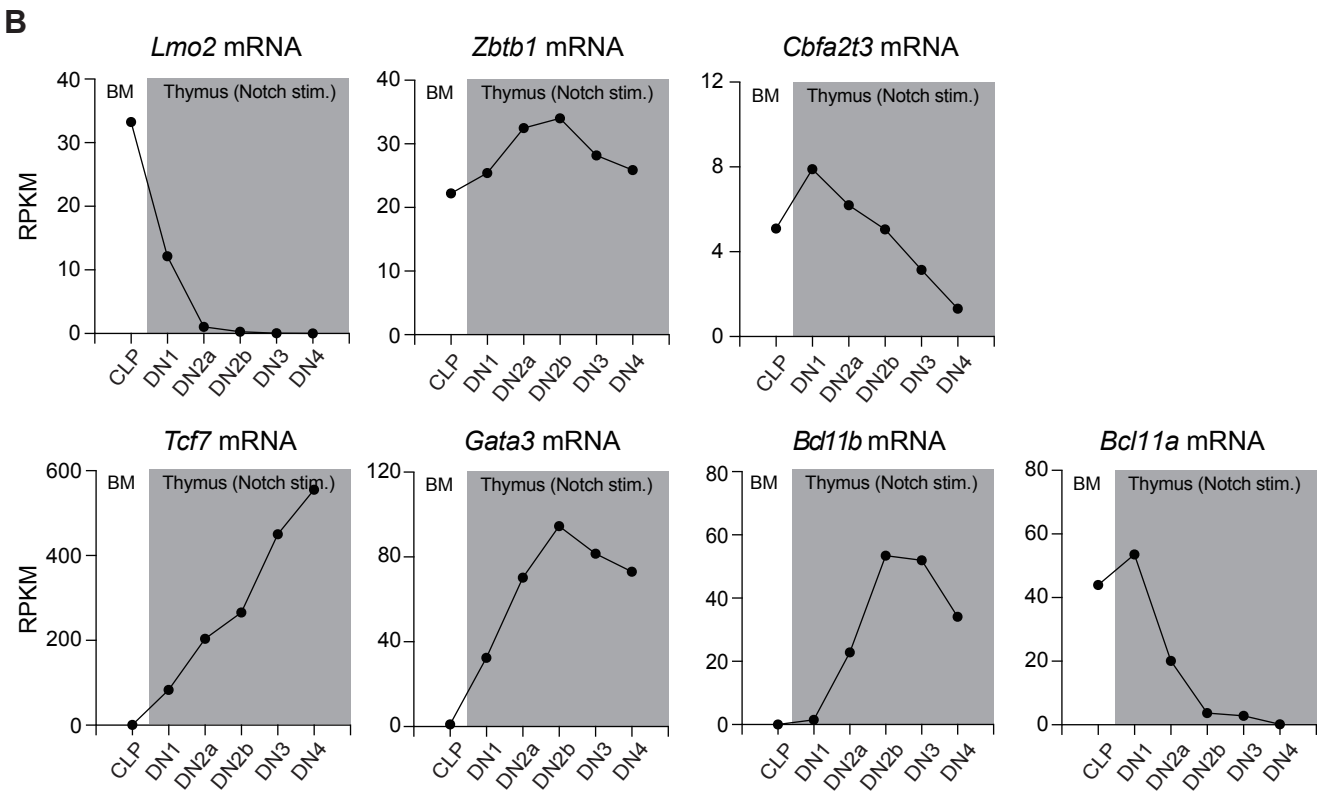

Supplement: Figure S1 [file mmc4.pdf]

Supplemental Figure S2

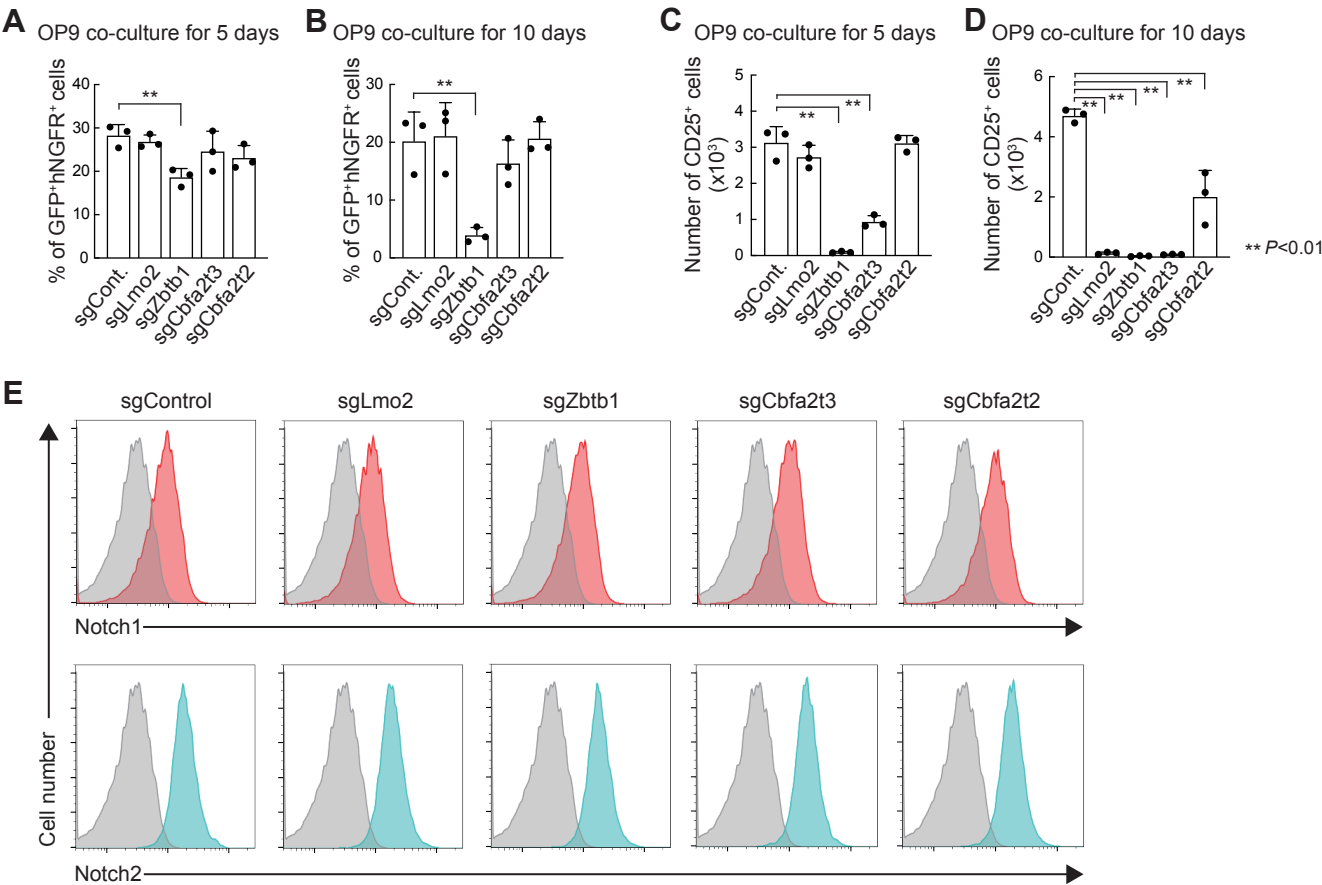

Supplement: Figure S2 [file mmc5.pdf]

Supplemental Figure S3

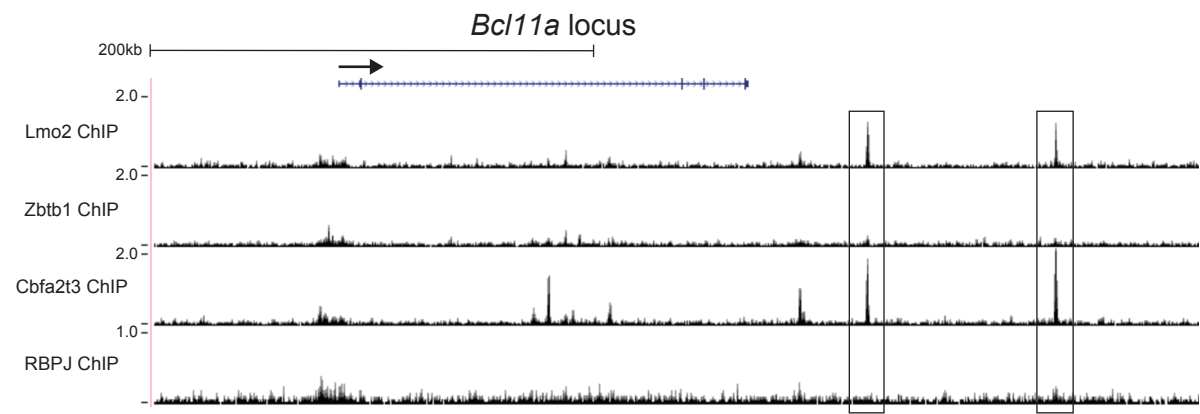

Supplement: Figure S3 [file mmc6.pdf]

Supplemental Figure S4

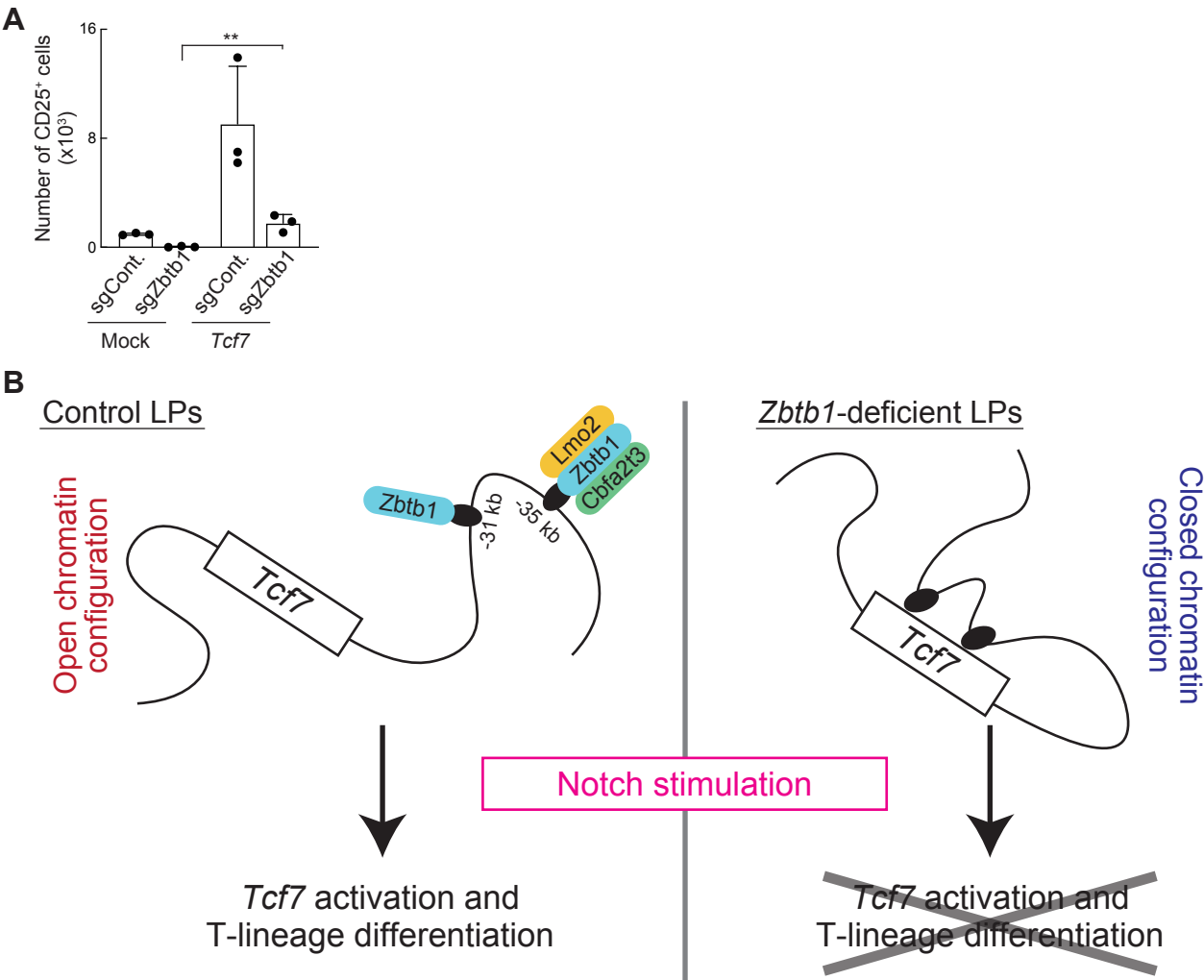

Supplement: Figure S4 [file mmc7.pdf]
